# Dose-Dependent PFESA-BP2 Exposure Increases Risk of Liver Toxicity and Hepatocellular Carcinoma

**DOI:** 10.3390/cimb47020098

**Published:** 2025-02-05

**Authors:** Grace Kostecki, Kiara Chuang, Amelia Buxton, Sivanesan Dakshanamurthy

**Affiliations:** 1 Yale College, Yale University, New Haven, CT 06520, USA; 2 College of Human Ecology, Cornell University, Ithaca, NY 14853, USA; 3 Department of Biomedical Engineering, College of Engineering, University of Maine, Orono, ME 04469, USA; 4 Department of Oncology, Lombardi Comprehensive Cancer Center, Georgetown University Medical Center, Washington, DC 20007, USA

**Keywords:** per- and polyfluoroalkyl substances, PFESA-BP2, Nafion by-product 2, PFESA-BP2 exposure, hepatotoxicity, hepatocellular carcinoma, PFESA-BP2-induced tumor biomarkers, mechanisms

## Abstract

Per- and polyfluoroalkyl substances (PFASs) are persistent and highly bioaccumulative emerging environmental contaminants of concern that display significant toxic and carcinogenic effects. An emerging PFAS is PFESA-BP2, a polyfluoroalkyl ether sulfonic acid found in drinking water and the serum of humans and animals. While PFESA-BP2-induced liver and intestinal toxicity has been demonstrated, the toxicological mechanisms and carcinogenic potential of PFESA-BP2 have remained relatively understudied. Here, we studied how different doses of PFESA-BP2 affect gene activity related to liver toxicity and the risk of liver cancer such as hepatocellular carcinoma (HCC) in mice exposed to PFESA-BP2 once daily through oral gavage for seven days. An analysis of key hepatic pathways suggested increased risk of hepatotoxicity as a result of PFESA-BP2 exposure. Increased oxidative stress response was associated with all concentrations of exposure. Liver toxicity pathways, including PXR/RXR activation and hepatic fibrosis, showed dose-dependent alteration with activation primarily at low doses, suggesting an increased risk of hepatic inflammation and injury. Additionally, an analysis of carcinogenic and HCC-specific pathways suggested PFESA-BP2-induced risk of liver cancer, particularly at low doses. Low-dose PFESA-BP2 exposure (0.03 and 0.3 mg/kg-day) was associated with an increased risk of HCC carcinogenesis, as indicated by the activation of tumor-related and HCC-associated pathways. In contrast, these pathways were inhibited at high doses (3.0 and 6.0 mg/kg-day), accompanied by the activation of HCC-suppressive pathways. The increased risk of HCC development at low doses was mechanistically linked to the activation of signaling pathways such as HIF, EGF, NOTCH4, HGF, and VEGF. Biomarkers linked to liver cancer risk, prognoses, and diagnoses were also identified as a result of exposure. Overall, our findings on liver carcinogenic and hepatotoxic pathway activation patterns suggest that PFESA-BP2 increases the risk of liver toxicity and HCC development, particularly at low doses.

## 1. Introduction

Per- and polyfluoroalkyl substances (PFASs) are a class of highly persistent, widely used chemicals that require extensive periods of time for natural breakdown [1]. Consequently, PFASs have been found globally in the blood of humans and animals as well as in drinking water, fish, and soil, where they have been associated with adverse biological effects [2,3]. Despite predominant public and regulatory focus on a few common PFASs, the chemical class comprises millions of PFAS compounds according to the Organisation for Economic Co-operation and Development (OECD) [4]. Growing concerns over the potential health risks of PFAS exposure have led to the establishment of regulatory limits and advisory levels for select PFASs [5]. However, the vast majority of PFASs are both understudied and unregulated, despite their possible toxic and carcinogenic effects.

A new and relatively understudied PFAS is PFESA-BP2, a by-product of the manufacturing of Nafion membranes, commonly used in the chloralkali process [6]. To date, PFESA-BP2 has been identified in Cape Fear River and its surrounding locations in North Carolina [7,8,9,10,11]. However, given that Chemours, the leading manufacturer of Nafion membranes, operates Nafion production facilities in North Carolina, Delaware, and Villers-Saint-Paul, France, future investigations into those regions may reveal understudied effects of exposure [12,13,14]. Since 2012, the presence of PFESA-BP2 has been reported in laughing gull chicks, zebrafish, and striped bass. These species aid in transporting chemicals and inducing exposure to a wider population. Consequently, recent studies show the high bioaccumulation potential of PFESA-BP2, indicated by significantly elevated PFESA-BP2 serum concentrations in mice, even at low doses in drinking water [15,16]. Further, PFESA-BP2 has been identified in human serum near the Cape Fear River [7,8,9,10,11,16]. Although there is known human exposure, there is still little toxicological data on PFESA-BP2 [8,9]. Currently, few studies have demonstrated toxic effects such as liver and intestine dysfunction in zebrafish, mice, and rats following PFESA-BP2 exposure [17,18,19,20].

Additionally, despite associations with liver toxicity and altered liver function, the carcinogenic effects of PFESA-BP2 on hepatocellular carcinoma (HCC) have not been studied to date. HCC is the most common type of liver cancer in adults, and is the third leading cause of cancer-related deaths worldwide [21,22]. In addition to liver cirrhosis, hepatitis B virus, hepatitis C virus, and excessive alcohol consumption, PFAS exposure has emerged as a potential risk factor for HCC [23]. As PFAS tends to accumulate in protein-rich tissues, such as the liver, PFAS can impact hepatic lipids, amino acids, and glucose metabolism, which may increase risk of HCC [24,25]. Exposure to perfluorooctanesulfonic acid (PFOS), to which PFESA-BP2 is the most structurally similar, has been associated with changes to key metabolic pathways in the liver, such as glucose metabolism, bile acid metabolism, and the metabolism of branched-chain amino acids, and the alteration of these metabolic pathways increases risk of hepatotoxicity and HCC [24]. PFOS is also linked to insulin dysregulation and type II diabetes, other potential risk factors for HCC [24,25]. Significant associations have also been found between serum PFAS concentration and the incidence of HCC when rats were experimentally exposed to varying doses of PFAS, indicating a positive correlation between PFAS concentration and HCC [21].

Therefore, given the known carcinogenic and toxicological effects of other PFASs and the recurrent detection of PFESA-BP2 in drinking water, additional research is necessary. Specifically, more research is required to analyze the hepatotoxic and liver carcinogenic effects of PFESA-BP2, which remains understudied despite its associations with altered liver functioning [16]. The goal of this study is to evaluate the comprehensive effects of PFESA-BP2 exposure by a robust omics pipeline analysis [26,27,28]. We analyzed PFESA-BP2 exposure impacts on the altered gene expression and pathways involved in liver toxicity and liver cancer risk. Through a dose-dependent analysis of differentially expressed genes, hepatotoxic pathways, HCC pathways, and HCC biomarkers, this study seeks to understand the toxicological and carcinogenic potential of PFESA-BP2 in the liver. We ultimately demonstrated increased risk of liver toxicity and hepatocellular carcinoma development as a result of PFESA-BP2 exposure, specifically at lower doses.

## 2. Methods

### 2.1. RNA-Seq Analysis and Generation of Differentially Expressed Genes

**Figure 1:**
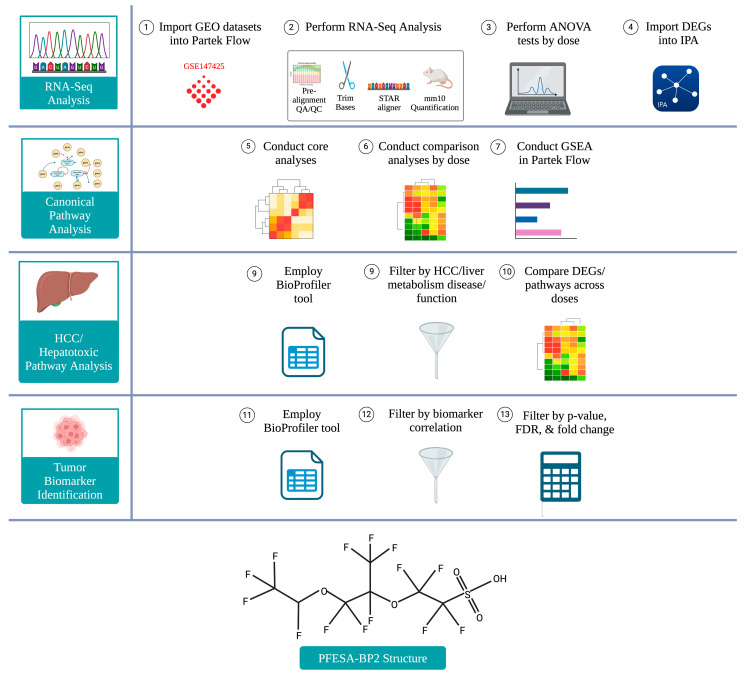
An illustration of the workflow to investigate the carcinogenic and toxic effects of PFESA-BP2 exposure on pathway and biomarker alterations. RNA-Seq Analysis: (1) We used the PFESA-BP2-exposed mouse liver dataset GSE147425 obtained from the GEO database. The dataset was input into Partek Flow using the accession numbers. (2) Unaligned reads were prepared for an analysis using pre-alignment QA/QC, trim bases, alignment using the STAR 2.8.7a index to the *Mus musculus* mm10 whole genome, and quantification to mouse model mm10 Ensembl transcripts, release 102. (3) Four ANOVA tests were conducted comparing each PFESA-BP2 dose (0.03, 0.3, 3.0, and 6.0 mg/kg-day) to the control (DMSO). The ANOVA tests produced DEGs based on dose, which were filtered by *p* < 0.05 and |log_2_ ratio| ≥ 1.5. (4) Differentially expressed genes (DEGs) identified by the Partek Flow ANOVA tests were imported into Qiagen IPA along with their fold change, FDR, and *p*-values. Canonical Pathway Analysis: (5) In IPA, core analyses were individually performed on DEGs from each PFESA-BP2 dose to yield activation/inhibition trends in canonical pathways. (6) A comparison analysis was performed to assess the activation/inhibition of canonical pathways and the mechanisms of DEGs between all PFESA-BP2 doses. (7) A Gene Set Enrichment Analysis (GSEA) was additionally performed in Partek Flow to generally determine the most strongly induced and repressed pathways in our dataset from a predefined set of global pathways. HCC/Hepatotoxic Pathway Analysis: (8) The BioProfiler tool was further employed to specifically analyze the effects of PFESA-BP2 exposure on liver toxicity and HCC development. (9) BioProfiler was used to filter for differentially expressed genes and pathways that were related to hepatocellular carcinoma (HCC) or liver toxicity under the “disease/function” tool. (10) Comparison analyses on these genes and pathways were used to assess differences in expression and activation/inhibition at different PFESA-BP2 doses versus DMSO. Tumor Biomarker Identification: (11) To identify tumor biomarkers in the PFESA-BP2-exposed dataset, DEGs were imported into the IPA BioProfiler tool. (12) DEGs were filtered by IPA into five biomarker categories: the diagnosis, prognosis, efficacy, response to treatment, and disease progression. (13) Only biomarkers with a *p*-value < 0.05, FDR < 0.05, and |fold change| ≥ 2 were included. Bottom: Structure of PFESA-BP2 (7H-Perfluoro-4-methyl-3,6-dioxaoctanesulfonic acid).

Dataset GSE147425 was selected after a comprehensive search for RNA-sequencing data related to PFESA-BP2 exposure in the Gene Expression Omnibus (GEO) database (National Center for Biotechnology Information, Bethesda, MD, USA) (Figure 1). This dataset analyzed liver samples from *Mus musculus* subjected to a once-daily gavage exposure to PFESA-BP2 for seven days. It included 100 liver samples from Balb/c mice (80 females and 20 males), distributed into five groups with 20 samples each: DMSO exposure (control), 0.03 mg/kg-day PFESA-BP2 exposure, 0.3 mg/kg-day PFESA-BP2 exposure, 3.0 mg/kg-day PFESA-BP2 exposure, and 6.0 mg/kg-day PFESA-BP2 exposure. Neither a chronic reference dose nor a maximum contaminant level has been established for PFESA-BP2 by the EPA. While all experimental doses used are larger than the chronic reference PFOS dose of 7.9 × 10^−9^ mg/kg-day, the four PFESA-BP2 exposure doses allow for a comprehensive dose-dependent analysis.

Raw data were then imported into Partek Flow 12.3.0 (Illumina, San Diego, CA, USA) for RNA sequencing using the GEO accession number. Low-quality bases were trimmed, and the trimmed reads were aligned to the *Mus musculus* mm10 whole genome using the STAR 2.8.7a index. The Quantification to Annotation Model task was performed on the aligned reads using the mm10 Ensembl transcripts, release 102. Four ANOVA tests were conducted, each comparing a different PFESA-BP2 dose to the DMSO control to generate *p*-values and log_2_ ratio values. After the RNA-sequencing analysis in Partek Flow, ANOVA data were filtered to identify differentially expressed genes (DEGs) for each PFESA-BP2 dosage group. Significance was determined by *p* < 0.05, and differential gene expression was classified using a log_2_ ratio cutoff of ±1.5. Log_2_ ratio values ≤ −1.5 were classified as downregulated, while values ≥ 1.5 were classified as upregulated [29].

### 2.2. Canonical Pathway Analysis

To determine the overall processes affected by PFESA-BP2 exposure, a Gene Set Enrichment Analysis (GSEA) was first performed to assess the activation/inhibition of predefined general global pathways. DEGs were imported into the “GSEA” function in Partek Flow to identify the most strongly activated and inhibited pathways as a result of PFESA-BP2 exposure according to their respective normalized enrichment scores. To identify the signaling genes responsible for these dysregulated biological processes affected by PFESA-BP2 exposure, an additional pathway analysis was conducted in Ingenuinity Pathway Analysis (QIAGEN Digital Insights, Aarhus, Denmark). DEGs (*p*-value < 0.05, |log_2_ ratio| ≥ 1.5) for each PFESA-BP2 dose were uploaded into the Qiagen Ingenuity Pathway Analysis (IPA) “core analysis” function for a canonical pathway analysis. Top canonical pathways were filtered by *p*-value and z-score (*p* < 0.05, |z-score| ≥ 1.5) and were categorized as activated (z-score ≥ 1.5) or inhibited (z-score ≤ −1.5) [30].

### 2.3. Analysis of Hepatotoxic and HCC-Associated Pathways Through IPA

For a liver-specific analysis on the dataset, DEGs and pathways were further filtered by association with liver toxicity or HCC. To determine DEGs and pathways related to liver toxicity, the IPA BioProfiler tool was utilized by selecting ‘liver toxicity’ in the ‘disease/function’ filter. To filter HCC-associated DEGs and pathways, the IPA BioProfiler tool was used by selecting ‘hepatocellular carcinoma’ in the ‘disease/function’ filter. Pathways with a z-score > 1.5 or <−1.5 were considered significantly altered by PFESA-BP2 and were further analyzed. Dose-dependent PFESA-BP2 response was determined by comparing log_2_ ratio values amongst doses for the DEGs and by comparing z-values amongst doses for the pathways. Lastly, mechanistic diagrams depicting the hypothesized role of PFESA-BP2 in activated/inhibited pathways were proposed based on our results and previous supportive studies.

### 2.4. Identification of Tumor Biomarkers Through IPA BioProfiler

Additionally, Qiagen IPA was employed to identify tumor biomarkers to investigate carcinogenic genes affected by PFESA-BP2 exposure. DEGs at each of the four doses were inputted into IPA with their associated fold change, FDR, and *p*-values and were subsequently analyzed using the BioProfiler tool in IPA. DEGs were filtered by BioProfiler based on known biomarker identity by integrating information from clinical trials and the literature. Biomarker candidates were further refined based on *p*-value, false discovery rate (FDR), and fold change (*p*-value < 0.05, FDR < 0.05, |fold change| > 2). BioProfiler additionally categorized the biomarkers by their biomarker classification (diagnosis, disease progression, efficacy, prognosis, and response to treatment) and associated cancer subtypes [31,32,33].

## 3. Results and Discussion

### 3.1. Differentially Expressed Genes from High PFESA-BP2 Exposure Reveal Associations with Hepatotoxicity and Liver Carcinogenesis

**Figure 2:**
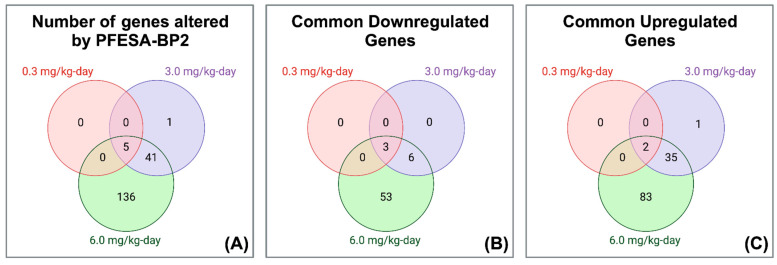
Significantly altered genes in liver cells following 0.03, 0.3, 3, and 6.0 mg/kg-day PFESA-BP2 exposures. Venn diagrams of (**A**) all significantly altered genes, (**B**) significantly downregulated genes, and (**C**) significantly upregulated genes using a ±1.5-z-score cutoff.

We identified differential gene expression at PFESA-BP2 exposures of 0.3, 3.0, and 6.0 mg/kg-day. Following a ±1.5 log_2_ ratio expression cutoff, none of the genes were significantly changed in the 0.03 mg/kg-day dose group. Five genes (two upregulated and three downregulated) were affected in the 0.3 mg/kg-day dose group. The 3.0 mg/kg-day dose group had 47 differentially expressed genes (38 upregulated and 9 downregulated) and the 6.0 mg/kg-day had 182 differentially expressed genes (120 upregulated and 62 downregulated) (Table 1; Figure 2A). Three downregulated genes (CYP2C40, CYP3A16, and FABP5) (Figure 2B, Table S1) and two upregulated genes (CYP2C55 and CYP2B10) (Figure 2C, Table S2) were common across the three highest doses.

Further, we identified 37 upregulated genes and 9 downregulated genes as a result of both 3.0 mg/kg-day and 6.0 mg/kg-day PFESA-BP2 exposure (Figure 2; Tables S3 and S4). The mean log_2_ ratio and average *p*-value for each gene were calculated by averaging the values of each dose. The top five over- and underexpressed genes (as identified by the largest and smallest average log_2_ ratio values, respectively) were chosen for a further analysis (Table 2). The top five upregulated genes (CYP2C44, GSTM3, CYP2B10, CYP4A14, and PRC1) revealed associations with liver cancer risk and liver injury. While CYP2C44 and GSTM3 have unclear relations to liver toxicity and carcinogenesis, CYP2B10 and CYP4A14 are directly linked to liver injury, inflammation, and lipid accumulation [34,35]. Hoflack et al. demonstrated that the induction of CYP2B10 in mice results in the activation of hepatic nuclear receptor constitutive androstane receptors, a promoter of hepatic tumors [35]. Zhang et al. found that CYP4A14 was overexpressed in liver samples of patients with nonalcoholic fatty liver disease [35]. In addition, Li et al. demonstrated that PRC1 is overexpressed in 28 cancer types including HCC and promotes cell proliferation and cell cycle progression [36]. Consistently, we found increased levels of CYP2B10, CYP4A14, and PRC1 that might suggest involvement in the induction of hepatic carcinogenesis and toxicity. We also found that the top five downregulated genes (ADAMTS6, BMF, CYP2C40, CYP3A16, EGR1) also point toward PFESA-BP2-induced risk of hepatic carcinogenesis and inflammation. While BMF is an apoptotic activator, ADAMTS6 and EGR1 are both tumor suppressor genes with links to liver cancer [37,38,39,40]. Specifically, Wang et al. demonstrated common decreased levels of EGR1 in HCC tissues from humans, rats, and mice [41]. Lastly, CYP3A16 is involved in lipid hydroxylation and breakdown in the liver [42]. The inhibition of HCC-suppressive genes further indicates that PFESA-BP2 exposure might be associated with risk of hepatic carcinogenesis.

### 3.2. GSEA Reveals PFESA-BP2-Induced Increase in Hepatic Cellular Metabolism

**Figure 3:**
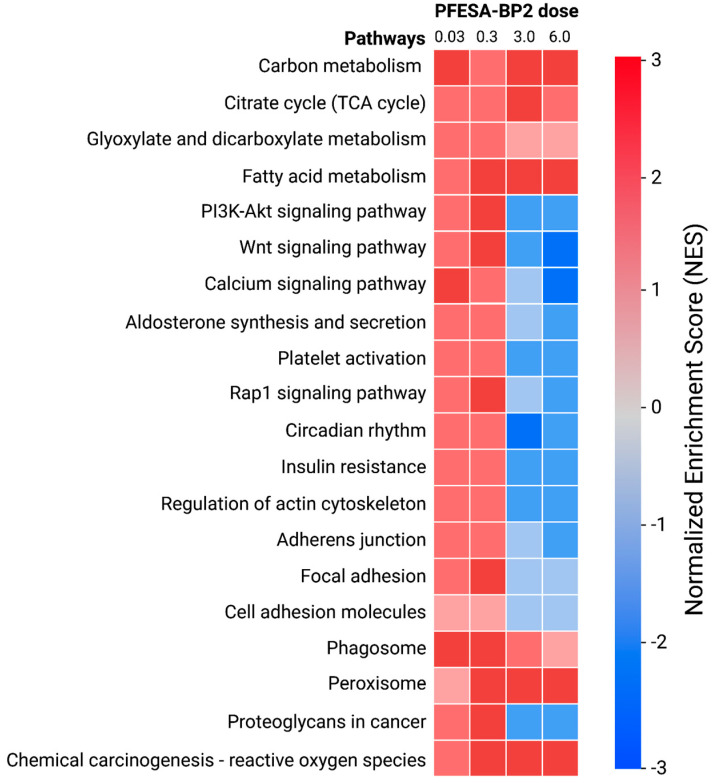
Top 20 most strongly activated/inhibited pathways in mouse liver cells in response to 0.03, 0.3, 3.0, and 6.0 mg/kg-day PFESA-BP2 exposures on the basis of the normalized enrichment score (NES) and *p*-value (*p* < 0.05) in the Gene Set Enrichment Analysis.

Upon identifying differentially expressed genes, we assessed general biological pathways affected by PFESA-BP2 exposure by conducting a Gene Set Enrichment Analysis (GSEA). A GSEA of the 20 most strongly activated/inhibited global pathways revealed common patterns of expression among genes with similar functions (Figure 3). Genes related to cellular metabolism, including those involved in ‘carbon metabolism’, ‘citrate cycle’, ‘glyoxylate and dicarboxylate metabolism’, and ‘fatty acid metabolism’, were among those most positively enriched at all doses of PFESA-BP2 exposure (Figure 3). The significant increase in cellular metabolism in the liver because of PFESA-BP2 exposure suggests that the liver may be a primary target of PFESA-BP2 toxicity.

### 3.3. PFESA-BP2 Exposure Increases Risk of Hepatic Inflammation and Toxicity

We further analyzed the effect of PFESA-BP2 exposure on liver toxicity, as we initially demonstrated an increase in cellular metabolism in the liver (Figure 3). We examined oxidative stress response and hepatotoxicity pathways to understand the genes and signaling pathways involved in PFESA-BP2-induced liver toxicity. In addition, we compared these effects at varying exposure doses to understand the dose dependence of PFESA-BP2.

#### 3.3.1. PFESA-BP2 Exposure Stimulates Oxidative Stress Response in the Liver

**Figure 4:**
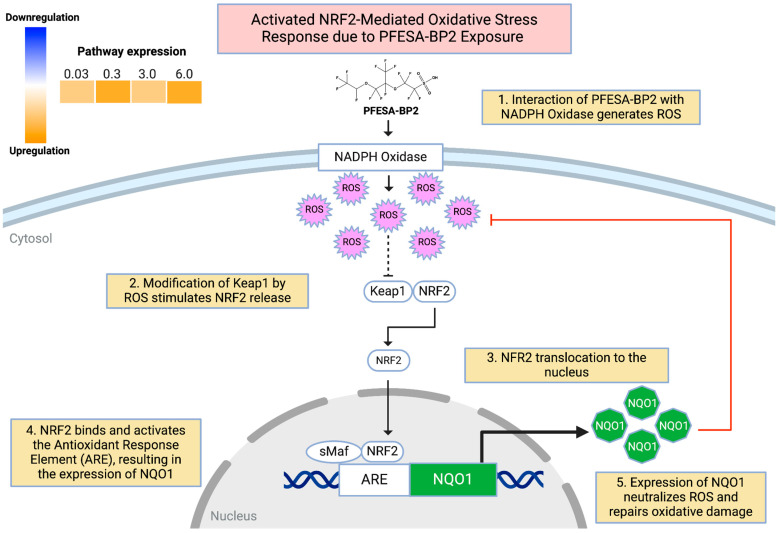
The proposed mechanism for the activation of the NRF2-mediated oxidative stress pathway in response to PFESA-BP2 exposure at all tested concentrations [49]. The interaction of PFESA-BP2 molecules with membrane-bound NADPH oxidase might have increased reactive oxygen species (ROS) production. The elevation in ROS levels might have activated NRF2-mediated oxidative stress, resulting in the overexpression of NQO1 (as indicated in green). This response facilitates the neutralization of ROS and oxidative damage repair.

A significant finding is that the NRF2-mediated oxidative stress response displayed activation in response to all concentrations of PFESA-BP2 exposure. Oxidative stress response is essential in cellular protection and maintenance of homeostasis against toxicity and oxidative stress in the liver [43,44]. We suggest that the activation might have occurred through PFESA-BP2 interaction with membrane-bound NADPH oxidases, generating reactive oxygen species (ROS) and inducing negative feedback to control ROS levels (Figure 4). As illustrated in Figure 4 ROS production induces a conformational change in cytosolic repressor protein KEAP1, resulting in the release of NRF2 from the KEAP1-NRF2 complex and translocation to the nucleus [45]. Activated oxidative stress response is observed in our dataset by the increased expression of protective enzyme NQO1 from NRF2 binding to and activating the antioxidant response element (ARE) and promoting NQO1 transcription. Higher NQO1 expression resulted in feedback inhibition by promoting the neutralization of ROS and repair of oxidative damage [46]. Further, these results demonstrate that PFESA-BP2 exposure at all doses increases the production of ROS, therefore increasing NRF2-mediated oxidative stress response as a form of negative feedback. Data further indicate that despite the increased production of ROS induced by PFESA-BP2 exposure, oxidative damage was minimized and cytotoxicity was prevented through the activation of oxidative stress response and NQO1. An increase in oxidative stress has also been observed with other PFASs, suggesting that PFESA-BP2 may generate and respond to oxidative stress through similar mechanisms [47,48]. Notably, PFESA-BP2 did not demonstrate a dose-dependent effect on the regulation of NRF2-mediated oxidative stress response; instead, activation occurred at all tested doses (Figure 4).

#### 3.3.2. PFESA-BP2 Exposure Increases Risk of Liver Toxicity

**Figure 5:**
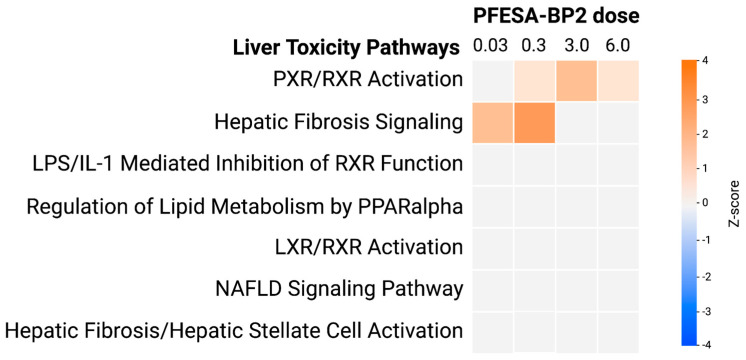
The analysis of 8 liver toxicity-related pathways across four doses of PFESA-BP2 exposure (in mg/kg-day) as generated by Qiagen IPA. The trend of pathway alteration is depicted in relation to the z-score, with z-scores ≤ −1.5 categorized as inhibition (blue) and z-scores ≥ 1.5 categorized as activation (orange). Results indicated PXR/RXR activation at the 0.3, 3.0, and 6.0 mg/kg-day doses. Hepatic fibrosis displayed activation at the lowest doses (0.03 and 0.3 mg/kg-day).

Along with oxidative stress response, we also aimed to determine the effect of PFESA-BP2 on pathways specifically involved in liver toxicity. An analysis of seven liver toxicity pathways as a result of PFESA-BP2 exposure demonstrated that most pathways remained unaltered except for ‘PXR/RXR activation’ and ‘Hepatic Fibrosis’ (Figure 5). The PXR/RXR activation was activated at the 0.3 mg/kg, 3.0 mg/kg, and 6.0 mg/kg doses but remained unaffected at the lowest dose, suggesting increased hepatic protection from potential damage through more clearance of xenobiotics like PFESA-BP2 [50]. In addition, hepatic fibrosis was activated at the lowest PFESA-BP2 doses but remained unaffected at the highest doses. This indicates progression to fibrosis as a result of chronic damage and inflammation from PFESA-BP2 exposure. Therefore, PFESA-BP2 exposure at different doses might have different implications for hepatic toxicity. However, the mechanisms require future experimentation and are further discussed in the following sections.

##### PFESA-BP2 Exposure Increases Hepatic Lipid Accumulation Through Increased PXR/RXR Activation

**Figure 6:**
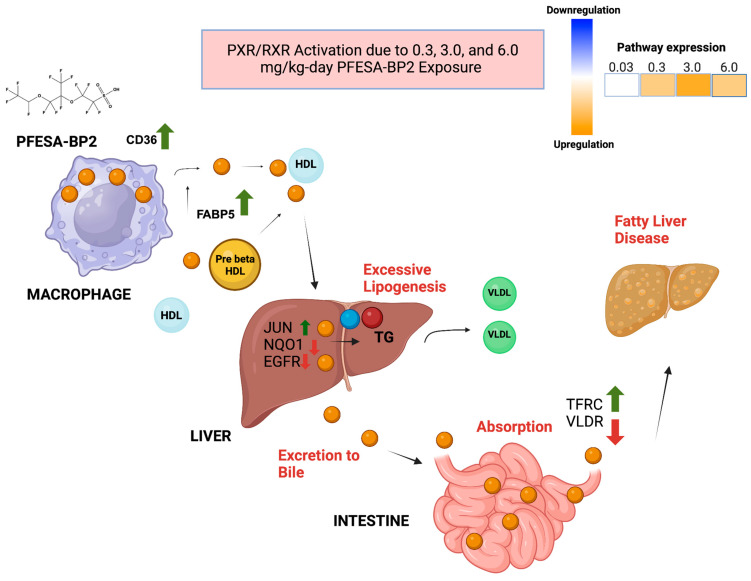
The proposed mechanism for PXR/RXR activation due to PFESA-BP2 exposure at 0.3, 3.0, and 6.0 mg/kg-day. The mechanism includes DEGs involved in liver function and lipid metabolism. The upregulated genes (CD36, FABP5, JUN, and TFRC) and downregulated genes (NQO1, EGFR, and VLDR) are critical to the PXR/RXR mechanism.

PXR/RXR activation at 0.3, 3.0, and 6.0 mg/kg-day was observed to be a result of the altered expression of key genes (Figure 5 and Figure 6). At the 0.3 mg/kg-day dose, FABP5 was significantly overexpressed, indicating lipid accumulation [49]. As the dose increased to 3.0 mg/kg-day, the expression levels of additional genes including CDK1 and CYP4A14 are increased, suggesting increased cell proliferation (CDK1) and hepatic inflammation (CYP4A14) [35,51]. At the highest dose of 6.0 mg/kg-day, a more complex pattern of gene expression was observed. Upregulated genes included CD36, CDK1, CYP4A11, CYP4A14, EPHX1, FABP5, JUN, LRG1, MYC, and TFRC. The high expressions of CD36 and JUN are particularly noteworthy, as they also point to increased lipid accumulation [52]. Conversely, several genes were downregulated at this dose, including ALDH1A1, EGFR, IGF1, LGALS1, NQO1, and VLDLR. The underexpression of NQO1, an enzyme that reduces oxidative stress, may suggest a faulty ability to manage reactive oxygen species that exacerbate liver toxicity [53]. The decreased levels of VLDLR demonstrate a disruption in the ability to reduce triglyceride levels, which could contribute to increased lipid accumulation [54]. The overall dose-dependent pattern of gene expression suggests that higher PFESA-BP2 doses correlate with PXR/RXR activation. Excessive lipogenesis, evidenced by the upregulation of CD36 and downregulation of VLDLR, indicates a potential mechanism for lipid accumulation in the liver directly from PFESA-BP2 exposure.

##### Low-Dose PFESA-BP2 Exposure Induces Hepatic Fibrosis Signaling

**Figure 7:**
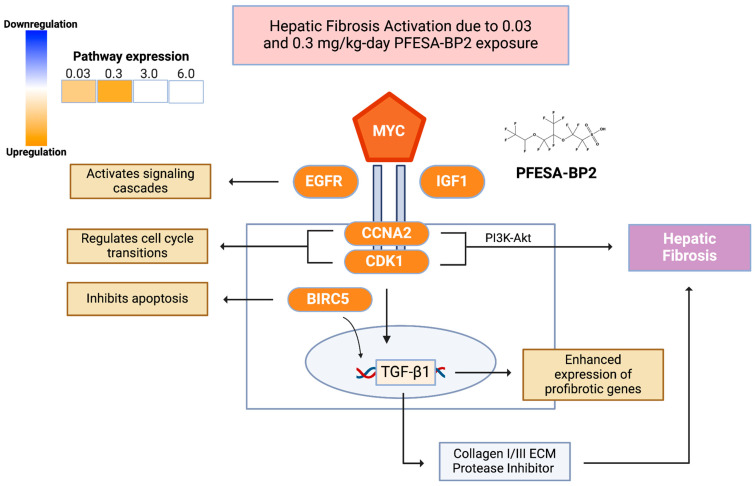
The proposed effect of low-dose PFESA-BP2 exposure on the hepatic fibrosis signaling pathway in the liver [59]. This figure depicts key genes involved in hepatic fibrosis, including MYC, EGFR, IGF1, CCNA2, CDK1, and BIRC5, which are all upregulated in response to PFESA-BP2 (indicated in orange). Therefore, PFESA-BP2-induced hepatic fibrosis might result in the activation of the PI3K-Akt pathway, increased collagen production, and ECM protease inhibition, key factors in fibrosis development.

We found activated hepatic fibrosis signaling at lower doses of 0.03 and 0.3 mg/kg-day PFESA-BP2 exposure (Figure 5 and Figure 7). At the 0.3 mg/kg-day dose, genes such as CYP2C18 and FABP5 were upregulated, while CYP2B6 was downregulated. Hepatic fibrosis activation at lower doses suggests a buildup of scar tissue in the liver as it attempts to repair damaged liver cells [55,56]. An increased expression of CYP2C18, predominantly found in the liver, indicates increased oxidation and metabolism in response to PFESA-BP2 exposure [57]. The overexpression of FABP5, a fatty acid-binding molecule and disease progression biomarker for liver cancer, also demonstrates lipid accumulation [49,58]. Therefore, while hepatic fibrosis may result from cell repair systems attempting to repair damage caused by lower-level PFESA-BP2 exposure, higher PFESA-BP2 doses such as 3.0 and 6.0 mg/kg-day exposures may be too toxic to induce the cellular repair that results in hepatic fibrosis.

### 3.4. PFESA-BP2 Exposure Increases Risk of HCC Development in a Dose-Dependent Manner

Building on our demonstrated hepatotoxic effect of PFESA-BP2 exposure, we investigated whether PFESA-BP2 additionally possesses possible carcinogenic potential in the liver. We analyzed pathways related to three categories: (1) the cell cycle, (2) general carcinogenesis, and (3) HCC association.

#### 3.4.1. PFESA-BP2 Exposure Increases Cell Cycle Progression

**Figure 8:**
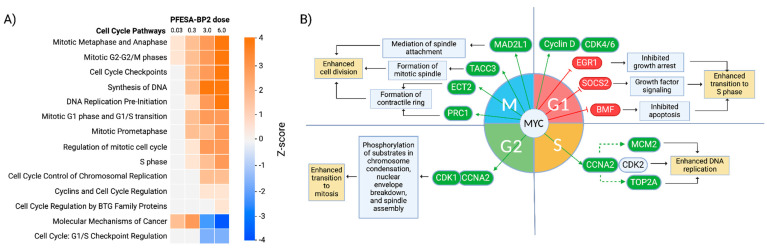
The analysis of cell cycle pathways and differentially expressed genes because of different levels of PFESA-BP2 exposure. (**A**) A heat map of cell cycle pathways across all 4 PFESA-BP2 doses (in mg/kg-day) as generated by Qiagen IPA. Pathways with z-scores ≤ −1.5 were categorized as inhibited (blue) and z-scores ≥ 1.5 as activated (orange). (**B**) Diverse mechanisms are considered for the involvement of transcription factor MYC in cell cycle activation as a result of PFESA-BP2 exposure. Proposed mechanisms involve the overexpression of genes (indicated in green) involved in DNA replication in the S phase, phosphorylation of substrates associated with transition to mitosis, mitotic spindle attachment and formation, and contractile ring formation. Other mechanisms also involve the inhibition of genes (indicated in red) in the G1 phase involved in growth arrest, growth factor signal inhibition, and apoptosis.

The pathways most significantly dysregulated due to PFESA-BP2 exposure were predominantly those related to the cell cycle, suggesting significant effects on risk of carcinogenesis. Results indicated the activation of many of these pathways across most PFESA-BP2 concentrations. Among the cell cycle-associated pathways generated by IPA, 14 exhibited activation or inhibition at one or more PFESA-BP2 concentrations (Figure 8A). Specifically, ‘Mitotic Metaphase and Anaphase’ and ‘Mitotic G2-G2/M Phases’ were consistently activated at all PFESA-BP2 concentrations. Half of the 14 pathways were activated at 0.3, 3.0, and 6.0 mg/kg-day (Figure 8A). PFESA-BP2 concentrations of 3.0 and 6.0 mg/kg-day were uniquely associated with the activation of more cell cycle pathways. Additionally, ‘Cell Cycle Regulation by BTG Family Proteins’ was activated exclusively at 6.0 mg/kg-day. Conversely, ‘Cell Cycle: G1/S Checkpoint Regulation’ was one of the only inhibited cell cycle pathways, observed at 3.0 and 6.0 mg/kg-day PFESA-BP2 exposures. Uniquely, Molecular Mechanisms of Cancer was activated at low PFESA-BP2 doses (0.03 and 0.3) and inhibited at high doses (3.0 and 6.0).

PFESA-BP2 exposure resulted in the constant overexpression of Myc, an oncogenic transcription factor that increases and decreases the levels of cell cycle genes at different cell cycle phases (Figure 8B) [60,61,62]. In the G1 phase, higher Myc levels were associated with increased CDK4, activating the transcription of genes essential for DNA synthesis and entry into the S phase. Simultaneously, Myc was associated with the downregulation of EGR1, SOCS2, and BMF, genes associated with growth arrest, growth factor signaling, and apoptosis, respectively. This suggests a faster transition into the S phase through the PFESA-BP2-induced upregulation of Myc. During the S phase, Myc upregulation was linked to higher levels of Cyclin A, which resulted in the phosphorylation and upregulation of helicase gene MCM2 and topoisomerase gene TOP2A, essential components of DNA replication. Through these mechanisms, PFESA-BP2 caused an increased replication of DNA during the S phase. In the G2 phase, Cyclin A activation was associated with higher levels of the CDK1/Cyclin A complex, resulting in an increased phosphorylation of substrates involved in chromosome condensation, nuclear envelope breakdown, and spindle assembly [63]. The increased activity of the CDK1/Cyclin A complex as a result of PFESA-BP2 exposure allows the cell to quickly and more efficiently prepare for cell division. Finally, increased cell division during the M phase was associated with the Myc-induced overexpression of MAD2L1, TACC3, ECT2, and PRC1, genes involved in the assembly of the mitotic spindle and contractile ring for proper chromosome alignment and cytokinesis. The activation of the cell cycle through the Myc-induced upregulation of key cell cycle genes because of PFESA-BP2 exposure has significant implications for cell cycle proliferation, resistance to apoptosis, and carcinogenesis. Therefore, further study is required to determine the mechanistic pathways through which PFESA-BP2 exposure causes the overexpression of Myc.

#### 3.4.2. Low-Dose PFESA-BP2 Exposure Increases Risk of Carcinogenesis in the Liver

**Figure 9:**
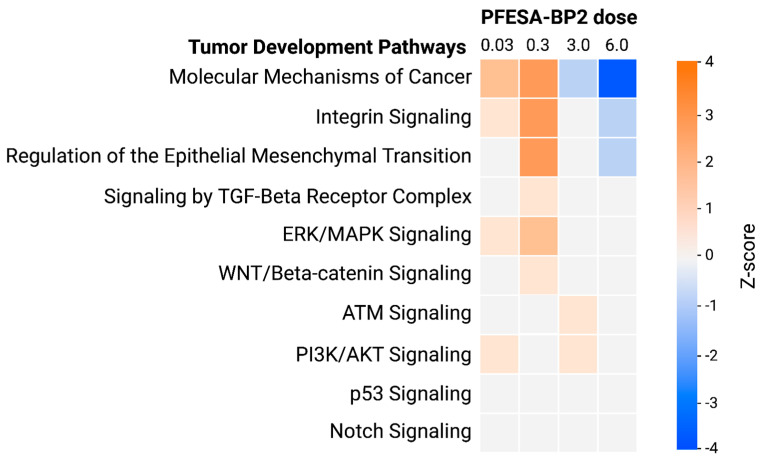
General carcinogenic pathways analyzed across 4 doses of PFESA-BP2 exposure (in mg/kg-day) as generated by Qiagen IPA. Pathways with z-scores ≤ −1.5 were categorized as inhibited (blue) and those with z-scores ≥ 1.5 were activated (orange).

An analysis of non-specific carcinogenic pathways demonstrated a common pattern: lower PFESA-BP2 doses (0.03 and 0.3 mg/kg-day) activated oncogenic pathways, while higher doses (3.0 and 6.0 mg/kg-day) inhibited these pathways and activated tumor-suppressive ones (Figure 9). Unlike the eight pathways that demonstrated these dose-dependent results, Notch signaling and p53 signaling were unaffected at all PFESA-BP2 doses. Uniquely, the PFESA-BP2 dose of 0.3 mg/kg-day was linked to the highest number of activated tumor-promoting pathways and was the only dose associated with the activation of TGF-Beta (TGF-β) receptor complex signaling and WNT/β-catenin signaling (Figure 9). Conversely, the PFESA-BP2 dose of 6.0 mg/kg-day was associated with the highest amount of inhibited tumor-promoting pathways, indicating the probable suppression of carcinogenesis. We found integrin signaling activation at 0.03 and 0.3 mg/kg-day and inhibition at 6.0 mg/kg-day (Figure 9). Activated integrin signaling increases the ability of integrins to bind to the extracellular matrix (ECM) and migrate as well as provides greater nutrients and oxygen to the growing tumor, contributing to metastasis and angiogenesis [64,65]. On the other hand, inhibited integrin signaling reduces the adhesion of integrins to the ECM, which can lead to tumor suppression and anoikis, programmed cell death when cells detach from the ECM [66,67,68]. Therefore, our results demonstrate that lower PFESA-BP2 concentrations (0.03 and 0.3 mg/kg-day) induce the tumor-promotive effects of integrin signaling, while a higher concentration of 6.0 mg/kg-day activates the tumor-suppressive properties of this pathway. ERK/MAPK signaling was activated at lower PFESA-BP2 doses (0.03 and 0.3 mg/kg-day), but remained unaffected at higher concentrations (Figure 9). Its activation upregulates transcription factors that drive the expression of genes involved in cell growth and downregulates pro-apoptotic proteins [69,70]. Consequently, this suggests that general tumor growth promotion through ERK/MAPK signaling is more likely to occur at lower PFESA-BP2 doses than at higher ones.

TGF-β signaling and WNT/Beta-catenin (WNT/β-catenin) signaling were both activated only at a PFESA-BP2 concentration of 0.3 mg/kg-day and unaffected at other doses (Figure 9). TGF-β signaling exhibits dual roles in all cancer subtypes, with tumor-suppressive properties in early stages and tumor-progressive properties in later stages. During early stages of cancer, it can induce cell cycle arrest by activating CDK–cyclin inhibitors and decreasing cyclin and CDK levels [71]. Given that CDKs and cyclins were normally expressed at the 0.3 mg/kg-day concentration, we cannot conclude that TGF-β signaling is tumor-suppressive as a result of PFESA-BP2 exposure. During later stages of cancer, tumor cells often become resistant to the suppressive nature of TGF-β signaling and the pathway switches to inducing Epithelial–Mesenchymal Transition (EMT), a process that enhances the migratory and invasive nature of tumor cells, promoting metastasis [72]. Exposure to PFESA-BP2 at 0.3 mg/kg-day resulted in a simultaneous activation of TGF-β signaling and EMT (Figure 9). In addition, higher WNT/β-catenin signaling is frequently associated with uncontrolled cell proliferation, resistance to apoptosis, and enhanced metastasis [73]. Consequently, the unique activation of both pathways at 0.3 mg/kg-day PFESA-BP2 suggests that this concentration may most effectively activate the tumor-promoting aspects of TGF-β and WNT/β-catenin signaling in all cancer subtypes. On the other hand, the activation of ATM signaling was observed at 3.0 mg/kg-day, demonstrating tumor-suppressive effects (Figure 9). Increased ATM signaling at 3.0 mg/kg-day PFESA-BP2 exposure suggests tumor-suppressive effects through the initiation of DNA-damage response, apoptosis, and cell cycle arrest by ATM [74]. ATM activation exclusively at this concentration suggests that 3.0 mg/kg-day is optimal for suppressing tumor progression through ATM.

Finally, PI3K/AKT signaling exhibited distinct results, demonstrating activation at only 0.03 and 3.0 mg/kg-day of PFESA-BP2 exposure (Figure 9). This indicates that these doses are most likely to activate the tumor-promotive aspects of this pathway through increased cell proliferation, reduced apoptosis, and enhanced angiogenesis [75,76]. However, given the observed activation at two non-adjacent doses (0.03 and 3.0 mg/kg-day), further research is necessary to determine the dose-dependent effects of PFESA-BP2 exposure on PI3K/AKT signaling.

#### 3.4.3. PFESA-BP2 Exposure Dysregulates HCC-Specific Pathways in a Dose-Dependent Manner

**Figure 10:**
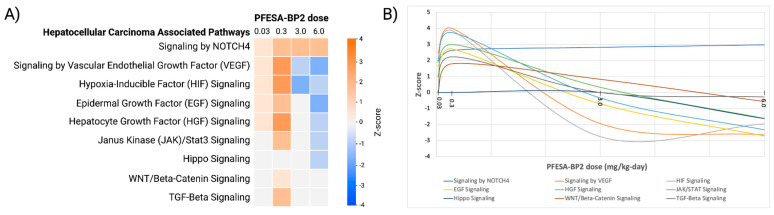
Analysis of 9 HCC-associated pathways across four doses of PFESA-BP2 exposure (in mg/kg-day) as generated by Qiagen IPA. (**A**) Heat map of HCC-associated pathways. They were classified by *p* < 0.05 and z-score: *z* ≤ −1.5 as inhibited (blue) and *z* ≥ 1.5 as activated (orange). (**B**) Dose response curve `12of z-scores of HCC-associated pathways upon varying PFESA-BP2 exposure.

After demonstrating alterations in non-specific carcinogenic pathways as a result of PFESA-BP2 exposure, we specifically analyzed HCC-associated pathways. Nine HCC-associated pathways were commonly activated at low PFESA-BP2 doses and inhibited at high doses (Figure 10). Others showed activation exclusively at low PFESA-BP2 doses (‘WNT/β-catenin signaling’ and ‘TGF-β signaling’) or inhibition at high PFESA-BP2 doses (‘Hippo signaling’). Notably, an exception to this pattern occurred with ‘signaling by NOTCH4’, which displayed activation at all PFESA-BP2 doses. Of the nine HCC-associated pathways, ‘Hippo signaling’ is recognized as HCC-suppressive, ‘signaling by NOTCH4’ and ‘TGF-β signaling’ have dual roles (either suppressive or promotive of HCC with context-dependent behavior), and the remaining are HCC-promotive. PFESA-BP2 exposure at 0.3 mg/kg-day resulted in the highest number of activated HCC pathways (eight) and 6.0 mg/kg-day exposure resulted in the highest number of inhibited ones (six). Among the eight activated pathways at the 0.3 dose, six are known HCC-promoting pathways, and two exhibit HCC-promoting or HCC-suppressive behavior depending on the cellular context. Of the six inhibited pathways at the 6.0 dose, five are HCC-promoting and one is tumor-suppressive. These findings are consistent with our analysis of non-specific carcinogenic pathways, in which the activation of tumor-promotive pathways at 0.3 mg/kg-day and inhibition of tumor-promotive pathways at 6.0 mg/kg-day were observed (Figure 9). Therefore, these data suggest that the PFESA-BP2 exposure dose at 3.0 mg/kg-day demonstrates the highest risk of HCC development, whereas a higher dose of 6.0 mg/kg-day is least promotive of HCC development, potentially due to its high toxicity.

##### Differential Regulation of HCC-Promotive Pathways at Low and High PFESA-BP2 Doses

The pro-proliferative growth pathways by VEGF, EGF, HGF, JAK/Stat3, and HIF commonly displayed activation at low PFESA-BP2 doses and inhibition at higher PFESA-BP2 doses (Figure 10). This specific pattern suggests that varying doses of PFESA-BP2 may elicit distinct molecular responses that could affect HCC pathogenesis differently. The activation of these signaling pathways has been associated with HCC progression, while inhibition has been correlated with tumor suppression. EGF signaling contributes to HCC growth through enhanced metastasis and increased tumor aggression, HIF signaling enhances tumor blood supply in low-oxygen environments, JAK/Stat3 signaling promotes tumor proliferation in inflammatory conditions, and VEGF and HGF signaling are more involved in promoting angiogenesis [77,78,79,80,81,82]. Therefore, the findings demonstrate that exposure to lower PFESA-BP2 doses may enhance the tumor-promotive properties of these signaling pathways, while exposure to higher doses may suppress them.

**Figure 11:**
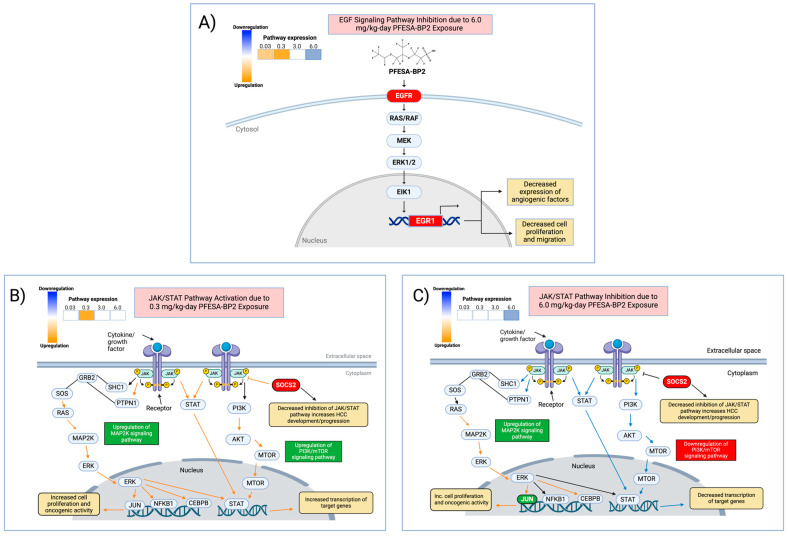
Hypothesized mechanistic diagrams for the effect of PFESA-BP2 exposure on EGF signaling and JAK/STAT signaling based on previous supportive studies. (**A**) Our proposed mechanism for the inhibition of EGF signaling at 6.0 mg/kg-day PFESA-BP2 exposure [84]. Inhibition occurred in association with the underexpression of core genes EGFR and EGR1 (depicted in red). (**B**) Our hypothesis for the mechanism by which PFESA-BP2 exposure at 3.0 mg/kg-day significantly altered genes and downstream regulators within JAK/STAT signaling [85]. The overall pathway was activated at 0.3 mg/kg-day, increasing the activation of STAT and the transcription of target genes. The two downstream oncogenic signaling pathways, MAP2K and PI3K/Akt/mTOR, were activated (depicted in green), increasing cell proliferation and oncogenic activity. The downregulation of SOCS2 (depicted in red) decreases the inhibition of JAK/STAT signaling, increasing its activation and risk of HCC development. (**C**) Our proposed mechanism for JAK/STAT signaling inhibition by PFESA-BP2 exposure at 6.0 mg/kg-day, decreasing the activation of STAT and the transcription of target genes [86]. Downstream MAP2K and PI3K/Akt/mTOR signaling was activated and inhibited, respectively. Increased MAP2K signaling activates genes involved in transcription. As a result, the overexpression of JUN, a key transcription factor in JAK/STAT signaling, increases cell proliferation and oncogenic activity. Reduced PI3K/Akt/mTOR signaling decreases the activation of STAT and the transcription of target genes. The downregulation of SOCS2 decreases the inhibition of JAK/STAT signaling, increasing the activation of the pathway and risk of HCC development.

Altered regulation likely occurred through different mechanisms, demonstrated by the differential expression of core components of each pathway. The activation of the HCC-associated pathways at low PFESA-BP2 exposure was not associated with the differential expression of any key genes within their pathways. Therefore, future experimentation is required to mechanistically explain the heightened risk of HCC development at low-dose exposure. However, the differential expression of key pathway genes at high-dose exposure may provide mechanistic clues for understanding pathway inhibition at high PFESA-BP2 exposure. In EGF signaling, exposure to higher PFESA-BP2 concentrations resulted in the downregulation of the key gene EGFR. This led to the decreased expression of EGR1, causing lower levels of angiogenic factors, cell proliferation, and migration (Figure 11A) [39]. Additionally, JAK/Stat3 signaling dysregulation was also associated with differentially expressed genes. PFESA-BP2 exposure at 0.3 mg/kg-day resulted in the activation of the pathway and decreased levels of SOCS2, a negative regulator of JAK/STAT signaling (Figure 11B). The underexpression of SOCS2 decreases its ability to negatively regulate JAK/STAT signaling, preventing the oncogenic pathway from being inhibited. PFESA-BP2 exposure at 6.0 mg/kg-day resulted in JAK/STAT signaling inhibition, JUN upregulation, and the downregulation of SOCS2 (Figure 11C). An increased activation of JUN, a transcription factor that positively regulates cell proliferation, is likely associated with carcinogenesis and could contribute to HCC risk [83]. Conversely, since SOCS2 functions as an inhibitor of JAK/STAT signaling, the lower levels of SOCS2 by PFESA-BP2 exposure reduce the inhibition of the pathway, potentially reducing its tumor-suppressive effects.

However, none of the core genes involved in VEGF signaling (VEGFR1, VEGFR2, ERK, AKT, PI3K), HGF signaling (HGF, MET, GRB2, PIK3K, AKT), or HIF signaling (HIF1*α*, HIF2*α*, HIF3*α*, ARNT, and ARNT2) were affected at any PFESA-BP2 dose. In addition, the differential expression of core genes within activated HCC-associated pathways did not occur at low PFESA-BP2 doses. Therefore, these alterations from PFESA-BP2 exposure might not be primarily due to transcriptional changes in significant genes but rather driven by post-translational modifications, protein stabilization, or changes in upstream signaling. Future research is required to further elucidate the different mechanisms of activation and inhibition at low and high PFESA-BP2 dose exposures.

##### Only Highest PFESA-BP2 Dose Exposure Alters Hippo Signaling

PFESA-BP2 exposure at a dose of 6.0 mg/kg-day resulted in the inhibition of Hippo signaling. In the liver, Hippo signaling activation suppresses hepatocyte proliferation and survival and is therefore associated with HCC suppression [87]. Inhibition results in an increased transcription of target genes that promote cell proliferation and hepatocyte proliferation, increasing the risk of HCC development [87,88]. The alteration of Hippo signaling at only 6.0 mg/kg-day suggests that higher PFESA-BP2 doses may be required to reduce the tumor-suppressive effects of the pathway and induce tumor-promotive properties.

##### Regulation of HCC-Associated Pathways by PFESA-BP2 vs. Other PFAS Chemicals

After investigating the risk of PFESA-BP2 exposure on HCC development, we assessed the regulation of HCC-associated pathways in comparison to other PFAS chemicals. Due to a lack of the literature linking signaling by HIF, NOTCH4, and JAK/STAT with PFAS, we were unable to perform comparative analyses. However, much of the current research on the correlation between other PFASs and EGF, Hippo, VEGF, HGF, TGF-β, and WNT/β-catenin signaling contradicts our results demonstrated with PFESA-BP2. For EGF signaling, Dunder et al. demonstrated that levels of EGFR were positively associated with exposure to PFOS, PFOA, and PFHxS, the opposite of which was shown in our results (Figure 6) [89]. Additionally, Clark et al. showed that PFOA exposure resulted in the activation of Hippo signaling in ovarian tissue, which contradicted the inhibition that occurred at 6.0 mg/kg-day PFESA-BP2 exposure (Figure 10A) [86]. In research by Forsthuber et al. and Salihovic et al., VEGF and HGF signaling were both shown to be negatively associated with PFOS and PFHxS, L-PFOS, PFOA, PFNA, and PFDA, respectively [90,91]. Our findings similarly showed the inhibition of these pathways at higher PFESA-BP2 concentrations; however, others did not share our unique pattern of activation at low PFESA-BP2 concentrations and inhibition at high concentrations (Figure 10). Moreover, TGF-β signaling, which displayed inhibition at only a PFESA-BP2 dose of 0.3 mg/kg-day, was otherwise shown by Rashid et al. to be increasingly activated with increased PFOA exposure in kidney tissue (Figure 10A) [92]. Lastly, Tsang et al. demonstrated decreased WNT/β-catenin signaling in endometrial cells upon PFOS exposure, contrasting from the activation demonstrated at a PFESA-BP2 dose of 0.3 mg/kg-day in our dataset (Figure 10A) [93]. Therefore, our findings suggest that PFESA-BP2 interacts with these pathways differently than other PFAS molecules.

### 3.5. Identification of Tumor Biomarkers Reveals Association with Breast and Liver Cancer Risk

**Figure 12:**
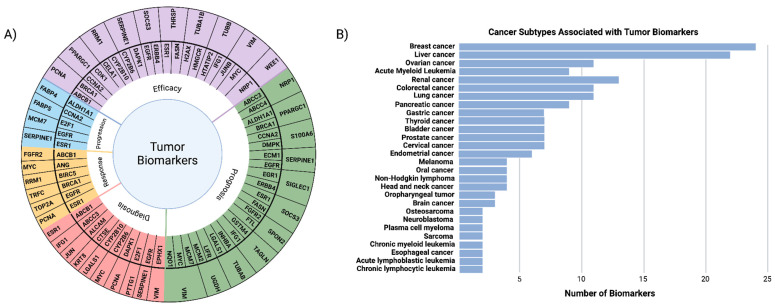
The identification of tumor biomarkers resulting from PFESA-BP2 exposure. (**A**) Specific biomarkers assessed in this study across the following categories: the diagnosis, efficacy, prognosis, progression, and response. (**B**) Frequency of cancer subtypes associated with the 62 tumor biomarkers identified from PFESA-BP2 exposure, with breast cancer and liver cancer showing the highest associations.

After assessing HCC-associated and general carcinogenic pathways affected by PFESA-BP2 exposure, we sought to identify specific carcinogenic genes affected. Using the identified DEGs as a result of PFESA-BP2 exposure, we identified 62 tumor biomarkers using the IPA Biomarker filter (Table S5). Of those identified, three were correlated with lower doses of PFESA-BP2 exposure, while the overwhelming majority were correlated with higher doses. Breast cancer (24 biomarkers) and liver cancer (22 biomarkers) were most frequently associated with the biomarkers (Figure 12). Despite limited research on the correlation of PFESA-BP2 with breast and liver cancers, other PFAS exposures including PFOS, PFOA, and PFHxS have known association with both cancer subtypes [25,28,94,95,96]. The majority of biomarkers were diagnosis biomarkers (34 biomarkers), efficacy biomarkers (29 biomarkers), or prognosis biomarkers (20 biomarkers). A high prevalence of diagnosis and prognosis biomarkers as a result of PFESA-BP2 exposure could suggest that it may alter critical biological pathways or processes that contribute to the development and worse prognosis of certain cancers [23,97]. Additionally, the presence of many efficacy biomarkers demonstrates that these 29 biomarkers, which are differentially expressed as a result of PFESA-BP2 exposure, could be used as drug targets for cancer therapeutics [23,97]. EGR1, MYC, and JUN are highly notable biomarkers that have been previously discussed due to their roles in liver carcinogenesis. These results suggest that high-dose PFESA-BP2 exposure dysregulates the expression of carcinogenic genes, suggesting that it might be associated with the risk of developing liver cancer.

Our identification of tumor biomarkers as a result of PFESA-BP2 exposure has further implications. The high prevalence of diagnostic biomarkers suggests their potential for developing early diagnostic tools for hepatotoxicity or HCC risk in populations exposed to PFESA-BP2. While cross-species validation in human liver cells would be required in further experimentation to compare mouse and human biomarkers, the identified diagnostic biomarkers could be used as early indicators of liver toxicity and risk of HCC development in exposed individuals.

## 4. Strengths and Limitations

This study was carried out by collecting mouse liver dataset GSE147425 from the GEO database and analyzing the effects of 0.03, 0.3, 3.0, and 6.0 mg/kg-day PFESA-BP2 exposures. To the best of our knowledge, this is the first comprehensive study to investigate (1) the dose-dependent nature of PFESA-BP2 exposure and (2) the possible risks of PFESA-BP2 exposure on HCC development. Combining a gene expression analysis, pathway analysis, and tumor biomarker identification allowed us to gain a better understanding of the potential mechanisms underlying PFESA-BP2 carcinogenesis and toxicity. In particular, our focus on HCC risk as a result of PFESA-BP2 exposure, through the analysis of specific HCC pathways, revealed a previously unstudied association between PFESA-BP2 and HCC development.

However, several limitations persist in this study. Due to the use of only one PFESA-BP2-exposed dataset, our study can provide mechanistic clues for demonstrating PFESA-BP2 toxicity but does not capture the full effects of exposure. This study is an initial screening of important pathways affected by PFESA-BP2 exposure. Future study is required to validate key pathways and tumor biomarkers. In addition, as we only demonstrated the effects of four PFESA-BP2 doses, future experimentation with a greater range of doses will allow for a more specific dose-dependent analysis. Lastly, dataset GSE147425 included mostly female mouse samples subjected to only short-term PFESA-BP2 exposure. Therefore, follow-up experimentation is required to understand long-term PFESA-BP2 exposure and carcinogenic risk over time, as well as sex-specific differences.

## 5. Future Implications

Our findings suggest a uniqueness to PFESA-BP2, in which low-dose exposure (0.03 and 0.3 mg/kg-day) may pose a greater risk of hepatotoxicity and HCC development than higher doses. PFESA-BP2 has been detected in varying concentrations in different environments and animals: drinking water (~1000 ng/L), striped bass (1.03 ng/mL), laughing gull chicks (110 μg/kg), human serum in China (0.848 ng/mL), and human serum in North Carolina (16.9 ng/mL) [7,8,10,16,98,99]. However, all of these detected values are smaller than the PFESA-BP2 doses used in this study. Currently, there is no maximum contaminant level (MCL) for PFESA-BP2 set by the US-EPA, as it is not as widely present as other PFASs. However, since PFESA-BP2 is most structurally similar to PFOS, the MCL of PFOS (0.004 ng/mL) and current human serum levels of PFESA-BP2 (0.848 ng/mL and 16.9 ng/mL) can serve as reference points for determining the MCL of PFESA-BP2 [98,99,100]. Future study using real-world exposure concentrations is necessary to establish a chronic reference dose and maximum contaminant level for PFESA-BP2, informing regulatory guidelines by the US-EPA to mitigate the risk of liver toxicity. While this study does not directly propose these values, our results suggest the importance of assessing low-dose exposure in regulatory policies. This study also indicates that acute PFESA-BP2 exposure for only seven days, even at the lowest dose, can increase risk of liver toxicity and HCC development. The high bioaccumulation potential and environmental persistence of PFESA-BP2 suggest an even greater risk of chronic exposure. Costello et al. demonstrated a positive relationship between prolonged exposure of other PFASs and serum concentration, leading to increased risk of altered lipid metabolism, nonalcoholic fatty liver disease, and steatosis. Future study using long-term PFESA-BP2 exposure would be required to determine whether chronic PFESA-BP2 exposure demonstrates similar hepatotoxic effects. Further experimentation should focus on low-dose and long-term PFESA-BP2 exposure to assess chronic toxicity and provide recommendations to establish US-EPA regulatory standards for PFAS in drinking water.

## 6. Conclusions

This transcriptomic and genomic analysis of PFESA-BP2 exposure reveals the PFESA-BP2-induced dose-dependent dysregulation of key genes and pathways, specifically those related to hepatotoxicity and hepatocellular carcinoma (HCC). The alteration of liver toxicity pathways upon PFESA-BP2 exposure supports the growing literature that PFESA-BP2 exposure causes liver toxicity and provides insight into its mechanism, including increased NRF2 oxidative stress response, PXR/RXR activation, and hepatic fibrosis. In addition, an analysis of non-specific carcinogenic and HCC-specific pathways revealed that lower doses of PFESA-BP2 exposure (0.03 and 0.3 mg/kg-day) were correlated with the activation of tumor-promotive and HCC-promotive pathways, while higher doses (3.0 and 6.0 mg/kg-day) were associated with the inhibition of these pathways and the activation of tumor-suppressive and HCC-suppressive pathways. Lastly, 22 tumor biomarkers related to a liver cancer prognosis, diagnosis, and risk were identified in the exposed samples. These results suggest that low-dose PFESA-BP2 exposure may increase risk of liver toxicity and HCC development. This study uniquely combines transcriptomic, genomic, and biomarker analyses to assess PFESA-BP2-induced hepatotoxicity and risk of HCC development. Our findings reinforce evidence that PFESA-BP2 exposure has carcinogenic and toxic effects. However, it provides additional evidence on dose dependence in the liver, with results indicating that low-dose rather than high-dose exposure might increase risk of general tumor and HCC development.

## Figures and Tables

**Table 1:** Number of genes significantly altered by PFESA-BP2 exposure at different doses, using ±1.5 log_2_ ratio cutoff relative to unexposed samples.

Exposure	Upregulated	Downregulated
0.03 mg/kg-day	0	0
0.3 mg/kg-day	2	3
3.0 mg/kg-day	38	9
6.0 mg/kg-day	120	62

**Table 2:** The top 5 most significantly upregulated genes and downregulated genes across 3.0 mg/kg-day and 6.0 mg/kg-day PFESA-BP2 doses. These 10 genes were selected from a list of 37 common upregulated genes and 9 common downregulated genes across the two highest doses by having the highest average log_2_ ratio values (upregulation) and lowest average log_2_ ratio values (downregulation) and *p* < 0.05.

	*p*-Value for 3.0 mg/kg-Day	Log_2_ Ratio for 3.0 mg/kg-Day	*p*-Value for 6.0 mg/kg-Day	Log_2_ Ratio for 6.0 mg/kg-Day	Average *p*-Value	Average log_2_ Ratio
**Upregulated Genes**						
Cyp2c55	1.3494 × 10^−38^	5.49942044	5.1 × 10^−45^	6.65	6.7469 × 10^−39^	6.07471022
Gstm3	1.7561 × 10^−17^	4.9168889	8.41 × 10^−26^	6.39	8.7803 × 10^−18^	5.65344445
Cyp2b10	8.9576 × 10^−46^	3.88834182	1.05 × 10^−52^	4.71	4.4788 × 10^−46^	4.29917091
Cyp4a14	3.1072 × 10^−03^	3.08019952	1.74 × 10^−06^	4.43	1.55447 × 10^−03^	3.75509976
Prc1	1.0271 × 10^−08^	3.11662036	1.7 × 10^−12^	4.28	5.1365 × 10^−09^	3.69831018
**Downregulated Genes**						
Adamts6	9.1929 × 10^−05^	−1.568838	5.47018 × 10^−07^	−2.0248719	9.24756 × 10^−05^	−3.59371
Bmf	4.3447 × 10^−06^	−1.7893591	1.21484 × 10^−12^	−3.1173597	4.3447 × 10^−06^	−4.9067188
Cyp2c40	4.42809 × 10^−03^	−2.3517941	1.69481 × 10^−07^	−4.3073594	4.428263 × 10^−03^	−6.6591536
Cyp3a16	1.3199 × 10^−10^	−2.4715311	1.92432 × 10^−10^	−1.9226246	3.24426 × 10^−10^	−4.3941557
Egr1	1.2455 × 10^−08^	−2.8517665	1.59793 × 10^−10^	−3.6151756	1.26148 × 10^−08^	−6.4669421

## Data Availability

All data generated in this study are included in this article. Additional data generated are included in the Supplementary Material.

## Supplementary Materials

The following supporting information can be downloaded at: https://www.mdpi.com/article/10.3390/cimb47020098/s1.
