# Supplementary material for: Dose-Dependent PFESA-BP2 Exposure Increases Risk of Liver Toxicity and Hepatocellular Carcinoma
Source: Curr Issues Mol Biol. 2025 Feb 5;47(2):98. doi: 10.3390/cimb47020098 (PMC11854358; doi:10.3390/cimb47020098)
Supplement: Supplementary file 1 [file cimb-47-00098-s001.zip › cimb-3463650-supplementary.pdf]

Supplementary Table S1. 3 genes were commonly downregulated between 0.3, 3.0, and 6.0 mg/kg-day PFESA-BP2 exposures. Their $\log_2$ ratio values relative to the untreated samples and *p*-values were used to categorize downregulation ($\log_2$ ratio ≤ -1.5; $p < 0.05$).

**Gene name**	**0.3 mg/kg-day**	**3.0 mg/kg-day**	**6.0 mg/kg-day**
**CYP2C40**	-2.28	-2.35	-4.31
**CYP3A16**	-2.34	-2.47	-1.92
**FABP5**	-1.62	-1.56	-1.57

Supplementary Table S2. CYP2C55 and CYP2B10 were the two upregulated genes common between the 0.3, 3.0, and 6.0 mg/kg-day PFESA-BP2 exposures. Their corresponding $\log_2$ ratio values relative to the untreated samples and p-values were analyzed using ANOVA tests and used to categorize upregulation ($\log_2$ ratio ≥ 1.5, $p < 0.05$).

**Gene name**	**0.3 mg/kg-day**	**3.0 mg/kg-day**	**6.0 mg/kg-day**
**CYP2C55**	2.28	5.50	6.65
**CYP2B10**	2.51	3.89	4.71

Supplementary Table S3. 37 Upregulated genes were found to be common across 3.0 mg/kg-day and 6.0 mg/kg-day PFESA-BP2 doses. Upregulated genes were filtered by p-value < 0.05 and $\log_2$ ratio > 1.5.

**Upregulated Genes**	***p*-value for 3.0 mg/kg-day**	**$\log_2$ ratio for 3.0 mg/kg-day**	***p*-value for 6.0 mg/kg-day**	**$\log_2$ ratio for 6.0 mg/kg-day**	**Average *p*-value**	**Average $\log_2$ ratio**
4931406C07 Rik	1.1238E-07	1.50221343	1.75E-13	2.09	5.6188E-08	1.79610671
9130409I23 Rik	5.0334E-31	2.03874996	7.39E-41	2.72	2.5167E-31	2.37937498
Abcc4	0.00017214	1.94998204	4.41E-07	2.62	8.6289E-05	2.28499102
Adam11	1.3569E-08	1.98382645	6.67E-12	2.39	6.7876E-09	2.18691322
Birc5	8.2489E-23	2.53985286	4.69E-33	3.71	4.1244E-23	3.12492643
Ccn4	6.7627E-10	1.77142793	9.8E-14	2	3.3818E-10	1.88571397
Ccna2	5.7548E-25	2.65851427	1.36E-34	3.65	2.8774E-25	3.15425713
Cdca3	3.4103E-11	2.08845382	1.66E-19	2.99	1.7051E-11	2.53922691
Cdk1	2.2254E-20	2.37396863	2.41E-30	3.46	1.1127E-20	2.91698432
Ces2a	5.6722E-39	2.16663266	2.19E-53	3.17	2.8361E-39	2.66831633
Cyp26a1	4.0596E-13	2.49859955	1.19E-10	2.21	5.9703E-11	2.35429978
Cyp2b10	8.9576E-46	3.88834182	1.05E-52	4.71	4.4788E-46	4.29917091
Cyp2c54	4.3115E-05	1.51982178	0.00000262	1.89	2.2868E-05	1.70491089
Cyp2c55	1.3494E-38	5.49942044	5.1E-45	6.65	6.7469E-39	6.07471022
Cyp3a11	1.4351E-07	2.63765507	1.08E-10	3.34	7.181E-08	2.98882753
Cyp3a59	0.0121126	2.30980012	0.00033	2.89	0.0062213	2.59990006
Cyp4a10	0.00080723	1.53310738	2.57E-07	2.28	0.00040374	1.90655369
Cyp4a14	0.0031072	3.08019952	0.00000174	4.43	0.00155447	3.75509976
Ect2	3.9367E-14	2.41101076	2.7E-21	3.17	1.9684E-14	2.79050538
Fyb2	6.9907E-27	1.96842705	6.34E-37	2.59	3.4953E-27	2.27921352
Gm10447	1.2199E-09	1.55609954	6.88E-13	1.86	6.1032E-10	1.70804977
Gm14403	7.9366E-10	1.62057874	1.24E-14	2.2	3.9684E-10	1.91028937
Gm8834	0.00121569	2.30000817	9.55E-07	2.98	0.00060832	2.64000408
Gstm3	1.7561E-17	4.9168889	8.41E-26	6.39	8.7803E-18	5.65344445
Gstt3	4.4112E-12	1.50465319	7.55E-19	1.99	2.2056E-12	1.74732659
Hells	2.0675E-08	2.04081172	1.34E-12	2.71	1.0338E-08	2.37540586
Kif20a	1.9583E-12	2.57457483	9.94E-20	3.82	9.7915E-13	3.19728742

Knstrn	3.4026E-26	2.35860014	7.29E-36	3.28	1.7013E-26	2.81930007
Nek2	1.7754E-06	2.05173711	1.97E-11	2.82	8.8771E-07	2.43586856
Orm3	4.0378E-12	1.93714749	9.18E-19	2.68	2.0189E-12	2.30857374
Pctp	1.3932E-14	1.59081192	2.77E-28	2.6	6.9662E-15	2.09540596
Prc1	1.0271E-08	3.11662036	1.7E-12	4.28	5.1365E-09	3.69831018
Rrm2	3.5016E-16	2.18883904	3.2E-27	3.33	1.7508E-16	2.75941952
Sult2a1	0.04693666	1.69229243	0.0114	2.55	0.02916833	2.12114621
Sult2a7	2.1872E-07	1.50738037	1.35E-14	2.44	1.0936E-07	1.97369018
Top2a	7.7251E-23	2.46807275	5.13E-36	3.95	3.8625E-23	3.20903637
Tsku	4.5902E-19	3.08741004	1.54E-24	3.79	2.2951E-19	3.43870502

Supplementary Table S4. 9 genes were found to be commonly downregulated across both 3.0 mg/kg-day and 6.0 mg/kg-day PFESA-BP2 doses. Downregulated genes were filtered by *p*-value < 0.05 and $\log_2$ ratio < -1.5.

**Downregulated Genes**	***p*-value for 3.0 mg/kg-day**	**$\log_2$ ratio for 3.0 mg/kg-day**	***p*-value for 6.0 mg/kg-day**	**$\log_2$ ratio for 6.0 mg/kg-day**	**Average *p*-value**	**Average $\log_2$ ratio**
Adamts6	9.1929E-05	-1.568838	5.47018E-07	-2.0248719	9.24756E-05	-3.59371
Bmf	4.3447E-06	-1.7893591	1.21484E-12	-3.1173597	4.3447E-06	-4.9067188
Cyp2c40	0.00442809	-2.3517941	1.69481E-07	-4.3073594	0.004428263	-6.6591536
Cyp3a16	1.3199E-10	-2.4715311	1.92432E-10	-1.9226246	3.24426E-10	-4.3941557
Egr1	1.2455E-08	-2.8517665	1.59793E-10	-3.6151756	1.26148E-08	-6.4669421
Fabp5	3.4771E-10	-1.5589801	4.56367E-12	-1.5732104	3.52272E-10	-3.1321905
Plxnb1	6.9097E-07	-1.8651365	2.21451E-11	-2.7393261	6.90989E-07	-4.6044625
Socs2	2.7092E-13	-1.7463712	2.03655E-19	-2.9341624	2.70918E-13	-4.6805336
Ugt2b37	1.9022E-05	-1.5130725	6.83453E-08	-1.9989829	1.90902E-05	-3.5120554

Supplementary Table S5. 62 Tumor biomarkers shown in PFESA-BP2-exposed mice liver samples (as identified by both QIAGEN Ingenuity Pathway Analysis and Partek Flow). Biomarkers were filtered by $p < 0.05$, FDR < 0.05, and |fold change| > 2, and were organized by its associated PFESA-BP2 dose, biomarker category, and correlation with cancer subtypes.

Biomarker	Associated Dose	Type of Biomarkers	Top Cancers
ABCB1	6.0 mg/kg-day	Efficacy, Prognosis, Response to therapy	Acute myeloid leukemia, Gastric cancer, Melanoma, Osteosarcoma, Ovarian cancer, Renal cell carcinoma
ABCC3	6.0 mg/kg-day	Diagnosis, Prognosis	Neuroblastoma, Thyroid cancer, Liver cancer
ABCC4	3.0 mg/kg-day, 6.0 mg/kg-day	Diagnosis	Neuroblastoma
ALCAM	6.0 mg/kg-day	Prognosis	Ovarian cancer
ALDH1A1	6.0 mg/kg-day	Diagnosis, Disease progression	Breast cancer, Ovarian cancer, Clear cell renal cell carcinoma
ANG	6.0 mg/kg-day	Response to therapy	Ovarian cancer
BIRC5	3.0 mg/kg-day, 6.0 mg/kg-day	Response to therapy	Bladder cancer, Breast cancer, Colorectal cancer, Melanoma, Non-small cell lung carcinoma, Oral cancer, Oropharyngeal tumor, Pancreatic cancer, Plasma cell myeloma, Prostate cancer, Clear cell renal cell carcinoma, Liver cancer
BRCA1	6.0 mg/kg-day	Diagnosis, Efficacy, Response to therapy	Bladder cancer, Breast cancer, Endometrial cancer, Gastric cancer, Ovarian cancer, Peritoneal cavity cancer, Sarcoma, Liver cancer
CCNA2	3.0 mg/kg-day, 6.0 mg/kg-day	Diagnosis, Disease progression, Efficacy	Colorectal cancer, Non-Hodgkin lymphoma, Clear cell renal cell carcinoma
CDK1	3.0 mg/kg-day, 6.0 mg/kg-day	Efficacy, Prognosis	Acute myeloid leukemia, Chronic myeloid leukemia, Renal cell carcinoma, Liver cancer
CELA1	6.0 mg/kg-day	Efficacy	Pancreatic cancer
CTSE	6.0 mg/kg-day	Prognosis	Bladder cancer

CYP2B10	0.03 mg/kg-day, 0.3 mg/kg-day	Efficacy, Prognosis	Breast cancer
CYP2B6	0.03 mg/kg-day, 3.0 mg/kg-day, 6.0 mg/kg-day	Efficacy, Prognosis	Breast cancer
DAPK1	6.0 mg/kg-day	Efficacy, Prognosis	Gastric cancer, Non-Hodgkin lymphoma, Clear cell renal cell carcinoma, Liver cancer
DMPK	6.0 mg/kg-day	Diagnosis	Cervical cancer
E2F1	6.0 mg/kg-day	Disease progression, Prognosis	Bladder cancer, Ovarian cancer, Clear cell renal cell carcinoma, Liver cancer
ECM1	6.0 mg/kg-day	Diagnosis	Thyroid cancer, Liver cancer
EGFR	6.0 mg/kg-day	Diagnosis, Disease progression, Efficacy, Prognosis, Response to therapy	Bile duct cancer, Bladder cancer, Brain cancer, Breast cancer, Cervical cancer, Colon cancer, Colorectal cancer, Endometrial cancer, Endometrial carcinoma, Esophageal cancer, Gastric cancer, Head and neck cancer, Laryngeal cancer, Liver cancer, Non-small cell lung carcinoma, Oropharyngeal tumor, Ovarian cancer, Pancreatic cancer, Renal cell carcinoma, Skin cancer, Thyroid cancer
EGR1	3.0 mg/kg-day, 6.0 mg/kg-day	Diagnosis	Breast cancer, Pancreatic cancer, Clear cell renal cell carcinoma
EPHX1	3.0 mg/kg-day, 6.0 mg/kg-day	Prognosis	Acute myeloid leukemia, Lung tumor
ERBB4	3.0 mg/kg-day, 6.0 mg/kg-day	Diagnosis, Efficacy	Breast cancer,Gastric cancer, Non-small cell lung carcinoma
ESR1	6.0 mg/kg-day	Diagnosis, Disease progression, Efficacy, Prognosis, Response to	Acute myeloid leukemia, Breast cancer, Endometrial cancer, Nasopharyngeal carcinoma, Non-small cell lung carcinoma, Ovarian cancer, Liver cancer

		therapy	
FABP4	6.0 mg/kg-day	Disease progression	Melanoma, Liver cancer
FABP5	0.03 mg/kg-day, 3.0 mg/kg-day, 6.0 mg/kg-day	Disease progression	Oral cancer, Liver cancer
FASN	6.0 mg/kg-day	Diagnosis, Efficacy	Breast cancer, Non-small cell lung carcinoma, Clear cell renal cell carcinoma
FGFR2	6.0 mg/kg-day	Diagnosis, Response to therapy	Breast cancer, Endometrial cancer, Non-small cell lung carcinoma, Thyroid cancer
FTL	6.0 mg/kg-day	Diagnosis	Liver cancer
GSTM4	3.0 mg/kg-day, 6.0 mg/kg-day	Diagnosis	Breast cancer
H2AX	6.0 mg/kg-day	Efficacy	Acute lymphoblastic leukemia, Acute myeloid leukemia, Breast cancer, Chronic lymphocytic leukemia, Chronic myelomonocytic leukemia, Colorectal cancer, Endometrial cancer, Ovarian cancer, Pancreatic cancer, Liver cancer
HMGCR	6.0 mg/kg-day	Efficacy	Breast cancer
HTATIP2	6.0 mg/kg-day	Efficacy	Breast cancer
IGF1	6.0 mg/kg-day	Diagnosis, Efficacy, Prognosis	Bladder cancer, Brain cancer, Breast cancer, Cervical cancer, Colon cancer, Endometrial cancer, Ewing sarcoma, Head and neck cancer, Liver cancer, Non-Hodgkin lymphoma, Osteosarcoma, Plasma cell myeloma, Small cell lung carcinoma, Wilms tumor, Renal cell carcinoma
INHBA	6.0 mg/kg-day	Diagnosis	Colorectal cancer
JUN	6.0 mg/kg-day	Prognosis	Bladder cancer
JUNB	3.0 mg/kg-day, 6.0	Efficacy	Breast cancer

	mg/kg-day		
KRT8	6.0 mg/kg-day	Prognosis	Breast cancer
LGALS1	6.0 mg/kg-day	Diagnosis, Prognosis	Head and neck cancer, Pancreatic cancer, Clear cell renal cell carcinoma, Liver cancer
LIFR	6.0 mg/kg-day	Diagnosis	Thyroid cancer
MCM2	3.0 mg/kg-day, 6.0 mg/kg-day	Diagnosis	Cervical cancer, Lung tumor, Prostate cancer
MCM7	6.0 mg/kg-day	Diagnosis, Disease progression	Cervical cancer, Prostate cancer, Thyroid cancer, Liver cancer
MYC	6.0 mg/kg-day	Diagnosis, Efficacy, Prognosis, Response to therapy	Brain cancer, Breast cancer, Non-Hodgkin lymphoma, Non-small cell lung carcinoma, Oral cancer, Prostate cancer
NQO1	6.0 mg/kg-day	Diagnosis	Acute myeloid leukemia, Lung tumor, Liver cancer
NRP1	6.0 mg/kg-day	Diagnosis, Efficacy	Chronic lymphocytic leukemia, Colorectal cancer, Liver cancer
PCNA	6.0 mg/kg-day	Efficacy, Prognosis, Response to therapy	Acute lymphoblastic leukemia, Acute myeloid leukemia, Oropharyngeal tumor, Prostate cancer, Sarcoma, Renal cell carcinoma, Liver cancer
PPARGC1A	6.0 mg/kg-day	Diagnosis, Efficacy	Thyroid cancer
PTTG1	3.0 mg/kg-day, 6.0 mg/kg-day	Prognosis	Esophageal cancer
RRM1	6.0 mg/kg-day	Efficacy, Response to therapy	Acute myeloid leukemia, Pancreatic cancer
S100A6	6.0 mg/kg-day	Diagnosis	Breast cancer
SERPINE1	6.0 mg/kg-day	Diagnosis, Disease progression, Efficacy, Prognosis	Breast cancer, Colorectal cancer, Melanoma, Non-small cell lung carcinoma, Oral cancer, Ovarian cancer, Small cell lung carcinoma, Clear cell renal cell carcinoma
SIGLEC1	6.0 mg/kg-day	Diagnosis	Colorectal cancer, Head and neck

			cancer, Prostate cancer, Renal cancer
SOCS3	6.0 mg/kg-day	Diagnosis, Efficacy	Pancreatic cancer
SPON2	0.3 mg/kg-day, 6.0 mg/kg-day	Diagnosis, Prognosis	Gastric cancer, Liver cancer
TAGLN	6.0 mg/kg-day	Diagnosis	Cervical cancer
TFRC	3.0 mg/kg-day, 6.0 mg/kg-day	Response to therapy	Cervical cancer
THRSP	6.0 mg/kg-day	Efficacy	Breast cancer, Liver cancer
TOP2A	3.0 mg/kg-day, 6.0 mg/kg-day	Response to therapy	Breast cancer, Gastric cancer, Thymoma, Clear cell renal cell carcinoma, Liver cancer
TUBA1B	6.0 mg/kg-day	Diagnosis, Efficacy	Breast cancer
TUBB	6.0 mg/kg-day	Efficacy	Non-small cell lung carcinoma
UGDH	3.0 mg/kg-day, 6.0 mg/kg-day	Diagnosis	Prostate cancer
VIM	6.0 mg/kg-day	Diagnosis, Efficacy, Prognosis	Colorectal cancer, Mesothelioma,Ovarian cancer, Pancreatic cancer, Transitional-cell carcinoma
WEE1	6.0 mg/kg-day	Efficacy	Acute myeloid leukemia, Chronic myeloid leukemia
